# Cognitive diagnostic modelling in healthcare professions education: an eye-opener

**DOI:** 10.1007/s10459-022-10093-y

**Published:** 2022-02-24

**Authors:** Carlos Fernando Collares

**Affiliations:** 1 Department of Educational Development and Research, Faculty of Health, Medicine and Life Sciences, School of Health Professions Education (SHE), Maastricht University, Postbus 616, 6200 Maastricht, The Netherlands; 2 European Board of Medical Assessors, Edinburgh, UK; 3 Stichting Aphasia.help, Maastricht, The Netherlands

**Keywords:** Assessment, Clinical reasoning, Cognitive diagnostic modelling, Patient safety, Psychometrics, Standard setting

## Abstract

Criticisms about psychometric paradigms currently used in healthcare professions education include claims of reductionism, objectification, and poor compliance with assumptions. Nevertheless, perhaps the most crucial criticism comes from learners' difficulty in interpreting and making meaningful use of summative scores and the potentially detrimental impact these scores have on learners. The term "post-psychometric era" has become popular, despite persisting calls for the sensible use of modern psychometrics. In recent years, cognitive diagnostic modelling has emerged as a new psychometric paradigm capable of providing meaningful diagnostic feedback. Cognitive diagnostic modelling allows the classification of examinees in multiple cognitive attributes. This measurement is obtained by modelling these attributes as categorical, discrete latent variables. Furthermore, items can reflect more than one latent variable simultaneously. The interactions between latent variables can be modelled with flexibility, allowing a unique perspective on complex cognitive processes. These characteristic features of cognitive diagnostic modelling enable diagnostic classification over a large number of constructs of interest, preventing the necessity of providing numerical scores as feedback to test takers. This paper provides an overview of cognitive diagnostic modelling, including an introduction to its foundations and illustrating potential applications, to help teachers be involved in developing and evaluating assessment tools used in healthcare professions education. Cognitive diagnosis may represent a revolutionary new psychometric paradigm, overcoming the known limitations found in frequently used psychometric approaches, offering the possibility of robust qualitative feedback and better alignment with competency-based curricula and modern programmatic assessment frameworks.

## Foreword

Many questions currently plague those carefully reading the literature on assessment in healthcare professions education. How effective are scores in the prevention or timely detection of critical learning gaps? Why do students with similar test scores still perform so differently in practice? How appropriate is the current use of cut scores in cross-sectional summative assessments as the primary method for standard setting? To what extent do our current summative assessment practices still ensure patient safety? If the literature recommends more qualitative assessments, why are most pass/fail decisions still based on numbers instead of words? Is there a way to reconcile the quantitative and qualitative realms of assessment, close the gap and end this long-standing divide? Despite the expectations raised by asking these questions, I do not promise to have all the answers. Instead, I invite readers to learn more about a new psychometric paradigm called cognitive diagnostic modelling, which may help us get closer to the answer to these questions.

One of many potential explanations for the late application of cognitive diagnostic analyses in healthcare professions education may be teachers' fears and unfamiliarity with this novel approach. The aim of this paper is to demystify the use of cognitive diagnostic modelling and to illustrate its potential application. This paper also discusses the potential implications of cognitive diagnostic modelling for competency-based curricula and programmatic assessment and highlights areas for future research.

## Why do we need a new psychometric paradigm?

Classical test theory, generalisability theory, and item response theory have been the dominant psychometric paradigms for summative assessment in recent decades (Bloch & Norman, 2012; De Champlain, 2010). Classical test theory introduced the concepts of “true score” variance and “measurement error” variance. The separate estimation of these variances acknowledged the indirect nature of educational and psychological measurement. Generalisability theory enabled the estimation of different sources of measurement error. Task specificity was found to be the main source of unreliability, suggesting that competence is specific, not generic. Item response theory introduced a probabilistic approach to the estimation of examinees’ scores, measurement error and item difficulty parameters. Item response models allowed the estimation of the so-called “true scores” of test takers. Furthermore, item response theory also enabled the use of computerized adaptive testing, a technology that allows the dynamic selection of test items according to the ability of the test taker (Collares & Cecilio-Fernandes, 2019), and its application in progress testing (van der Vleuten et al., 2018).

However, despite these advances in psychometric methods, there has been an intense debate about the utility and appropriateness of these methods in healthcare professions education in the academic community, somewhat intensified by Schuwirth & van der Vleuten’s, 2006 paper. The authors correctly described several limitations of the current psychometric methods and of the psychometric rationale per se, having made a plea for the development of new psychometric models for use in educational assessment. Schuwirth and van der Vleuten (2006) turned their attention to the unresolved paradox caused by task specificity: the more we expand our test blueprint to convey higher levels of validity based on test content, the lower we can expect our reliability coefficients based on internal consistency to become. Considering this paradox, the authors ponder if our assessment tools should continue to assume unidimensionality, i.e., that each of the test items measures only a single latent trait throughout the test. In other words, the authors criticize the fact that summative scores may end up just “adding apples with oranges.” The assumption of unidimensionality may be challenging to hold with a high level of content heterogeneity or with items demanding a diverse myriad of cognitive processes to be solved. Items involving professionally authentic cognitive tasks that are challenging yet considerably different from other test items may appear in classical analyses as items with negative item-total correlations. In item response models, such items tend to have negative discrimination parameters and poor fit. The authors also noted that the current way in which knowledge, skills and attitudes are modelled as continuous latent variables culminates in a situation where much of the observed variance is discarded as measurement error. The authors’ criticism of such modelling is aligned with the observation that researchers tend to treat most of their variables of interest as quantitative by assumption. That is, researchers do not empirically demonstrate that these variables have an identical structure to real numbers (Michell, 1999). Moreover, item response models also need to comply with the assumption of local item independence, which conceives the items’ observed responses as exclusive reflective functions from the purported construct and not any other item or construct. This assumption can be even more problematic to hold in practice than is unidimensionality. Rigorous compliance with the local independence assumption may also limit the use of long, multistage, and therefore more realistic patient scenarios.

In addition to the arguments against the psychometric rationale described above, perhaps the most compelling argument against summative scores comes from the learners’ perspective. Learners may encounter practical difficulty in interpreting and making meaningful use of their scores, with a potentially detrimental impact on motivation, self-efficacy and ultimately performance. In other words, scores appear to be the most flawed form of feedback; yet, most undergraduate courses worldwide are still bound to their use (Cilliers et al., 2010). Furthermore, the impossibility of providing standardised assessments on top of Miller’s pyramid and the preference to change the focus from the assessment *of* learning using numbers (i.e., scores) to assessment *for* learning using words (i.e., qualitative feedback) made the use of nonpsychometric, qualitative validity frameworks necessary (Cook et al., 2016; Govaerts & van der Vleuten, 2013). The term “post-psychometric era” has become popular, and the debate about the appropriateness of using psychometric methods in healthcare professions education has continued to flourish, despite persistent calls for sensible use of modern psychometrics (Pearce, 2020; Schauber et al., 2018).

## Introducing cognitive diagnostic modelling

In the past few decades, a new psychometric paradigm that may provide an answer to the plea made by Schuwirth and van der Vleuten (2006) has emerged. *Cognitive diagnostic modelling*, also referred to in the literature as *diagnostic classification modelling*, comprises several latent variable models that measure large numbers of latent variables, which are set to be discrete. By discrete, we mean that the latent variables chosen to measure constructs of interest are no longer continuous, as in item response models, but categorical. In cognitive diagnostic modelling, latent variables are usually dichotomous (i.e., the test taker either has the attribute of interest or does not), but they can also be polytomous (i.e., the examinee is classified into a small number of ordinal categories of performance levels, as in a global rating scale with a limited number of categories).

Mathematically speaking, most models under the cognitive diagnosis umbrella stem from constrained log-linear analysis, which is used to model more than two categorical variables and may or may not assume the existence of a latent variable. This method can be construed as a restricted form of latent class analysis, which models the latent variables as categorical, with a limited number of discrete categories (von Davier, 2019). While item response theory models establish a formal relationship between the examinees’ ability level and their probability of success in the test items on a continuous scale, cognitive diagnosis models are used to estimate the relationship between the test takers’ cognitive attributes and the different attributes required to solve test items. The reader should note that the term “attribute” may refer to any cognitive process, skill, or competency deemed necessary constructs to correctly solve the problem.

In addition to the distinctive feature of having discrete latent variables, cognitive diagnostic modelling allows any item to reflect more than one latent variable concurrently, which previous psychometric paradigms did not allow. This property makes cognitive diagnosis modelling a feasible alternative approach that can encompass the multidimensionality known to exist within items, something intrinsic to certain well-known assessment tools, such as the situational judgement test (Garcia et al., 2014). The first instance of a peer-reviewed study using the cognitive diagnostic modelling approach in healthcare professions education was recently published in this journal (Schreurs et al., 2020). The authors looked for validity evidence based on the internal structure and response processes of two situational judgement tests used to select undergraduate medical students. Cognitive diagnosis was chosen because it was the only possible psychometric approach capable of addressing the typical within-item multidimensionality of the items from this assessment tool.

The origins of cognitive diagnostic modelling can be traced back to 1983 with the rule space method (Tatsuoka, 1983). The late professor Kikumi Tatsuoka noticed that several disciplines, including cognitive psychology, artificial intelligence and psychometrics, had been trying to develop methods for diagnosing students’ misconceptions, with mixed success. In this regard, she observed very accurately that binary scoring, with the representation of competence as a linear, continuous variable, which is still the most commonly used practice in testing, had fundamental limitations. A score of one is assigned even if the correct answer is achieved using the wrong cognitive process or “rule”. She also noticed that many erroneous “rules” could lead to a correct answer. Furthermore, a score of zero is assigned irrespective of the degree of misunderstanding resulting in the wrong answer. Tatsuoka was successful in demonstrating that students with similar scores may have an entirely different set of strengths and weaknesses by successfully predicting the likelihood of erroneous “rules” and diagnosing them correctly. Consequently, the proposition of representing competence as a multidimensional “space”, instead of a single continuous latent variable, could be seen as a more adequate approach.

Many different models have been developed under the label of cognitive diagnosis, and a discussion of each model is outside of the scope of this paper. We will only briefly introduce two such models, the *deterministic input, noisy “and” gate* (DINA) model and its generalised extension (G-DINA), the most used model in recent years. The DINA model assumes that examinees must possess all the skills required to solve a given problem accurately. In other words, the DINA model is a conjunctive model: a lack of one required attribute cannot be overcome by the presence of other attributes. The DINA model partitions examinees into two latent groups for each item. In one group, examinees have all required attributes to solve the item, and in the other group, the examinees lack at least one of the required attributes to solve the item. In the DINA model, examinees lacking one or more attributes are all constrained to have the same lower probability of success on the item. The G-DINA model relaxes this constraint, allowing different examinees from different latent classes (i.e., different combinations of attributes) to have different probabilities of success on an item, allowing a compensatory approach.

Two parameters are common to both the DINA and G-DINA models: the *guessing* parameter and the *slipping* parameter. Whereas the guessing parameter is a false-positive indicator, i.e., the probability of success when the examinee does not have the attributes, the slipping parameter can be seen as a false-negative indicator, i.e., the probability of failure when the examinee has the attributes (de la Torre & Minchen, 2014; Schreurs et al., 2020).

**Fig. 1:**
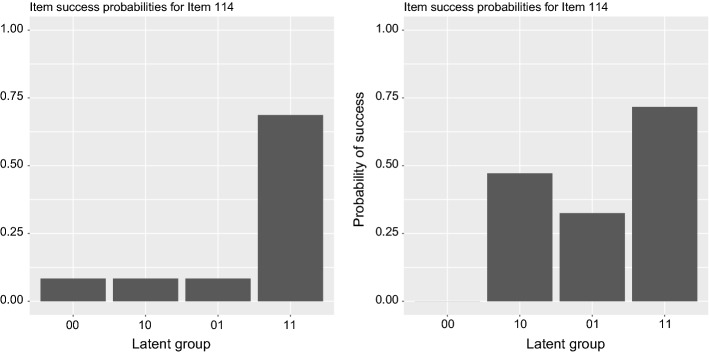
Probabilities of a correct answer on an item according to the presence of both attributes (11), one of them (01 or 10) or none of them (00), as estimated by the DINA (left) and G-DINA (right) models

Although the DINA model may seem overly restrictive due to its conjunctive nature and assumptions that may be challenging to hold, one may argue that a simpler model may be more appropriate, particularly in situations where test developers want to ensure the presence of all attributes or competencies. Figure 1 shows a comparison of how DINA and G-DINA estimate the probability of success differently for the same item, for which two different attributes are necessary for a correct answer.

A cardinal tenet that allows cognitive diagnostic analyses to be performed is the observation that a test taker who masters a certain number of skills or attributes will be capable of solving tasks that require these skills or attributes on a regular basis. The relationship between the performance of test takers on heterogeneous tasks and the multiple skills necessary to solve them must be systematically organized. Therefore, in a further development of the rule space method, Birenbaum et al. (1992) developed the Q-matrix: a way to arrange items according the necessary “rules” (i.e., cognitive attributes) needed to solve them (Birenbaum et al., 1992). The Q-matrix bears resemblance to the blueprint of a test, most frequently using binary indications only. Table 1 contains an example of a simple Q-matrix.

**Table 1:** Example of the proposed Q-matrix for the first ten items of a sample test destined to measure different applications of medical knowledge by medical students

Item number	Comprehension of normal processes	Comprehension of pathological processes	Clinical diagnosis	Treatment	Prevention	Interpretation and use of scientific evidence
1	1	0	0	0	0	0
2	1*	1	0	0	0	0
3	0	1	0	0	0	0
4	0	0	1	0	0	0
5	0	0	1	0	0	0
6	0	1*	1	1	0	0
7	0	0	1	1	0	0
8	1*	1*	0	0	1	0
9	0	0	0	1	0	1*
10	0	0	0	0	1	1

*Indicates a change in the designation of cognitive attributes suggested by the Q-matrix validation procedure

**Fig. 2:**
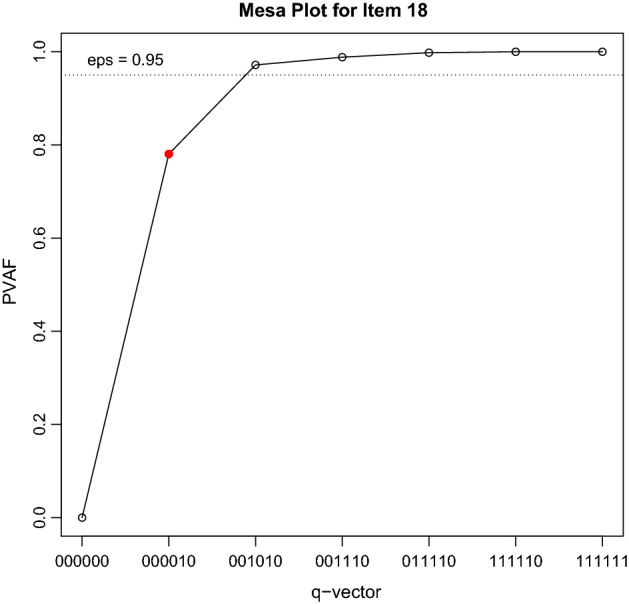
Example of a *mesa plot* used for Q-matrix validation using the PVAF method

The reader is correct to assume that misspecifications of the Q-matrix can have disastrous effects on the accuracy of cognitive diagnostic estimations. The most popular method for Q-matrix validation is the *percentage of variance accounted for* (PVAF). As a general rule, mesa plots can be interpreted similarly to scree plots generated in exploratory factor analysis and principal component analysis, popular methods for data reduction. The horizontal axis contains the different latent classes. The vertical axis informs us of the PVAF of each latent class. The latent class at the “edge of the mesa” tends to become the suggested set of skills in the Q-matrix validation. In other words, when inspecting a mesa plot, we must look for the most parsimonious latent class (i.e., the set of skills with the lowest number of attributes) that explains the largest amount of variance. Notably, any decisions regarding the correction of the Q-matrix should not be based solely on the results of a Q-matrix validation. Careful reflection by a multidisciplinary team of content experts and psychometricians is recommended for final decisions regarding any changes in the Q-matrix. Figure 2 shows an example of a *mesa plot*, in which it is possible to visualise the percentage of variance attributable to the different latent classes. The original set of cognitive skills determined by the Q-matrix accounted for approximately 80% of the variance. In the graph, a latent class with one additional attribute accounted for more than 95% of the variance.

Suppose this hypothetical item intended to measure only the "Prevention" attribute (identified as 000010), but a "hidden" cognitive task was necessary to solve the item: diagnosing hypertension. In this case, the Q-matrix validation procedure would have correctly identified the "hidden" cognitive process, suggesting the latent class 001010 for this item in the final Q-matrix with the inclusion of the attribute "Clinical diagnosis" (de la Torre & Akbay, 2019; de la Torre & Minchen, 2014; Ma, 2019).

**Fig. 3:**
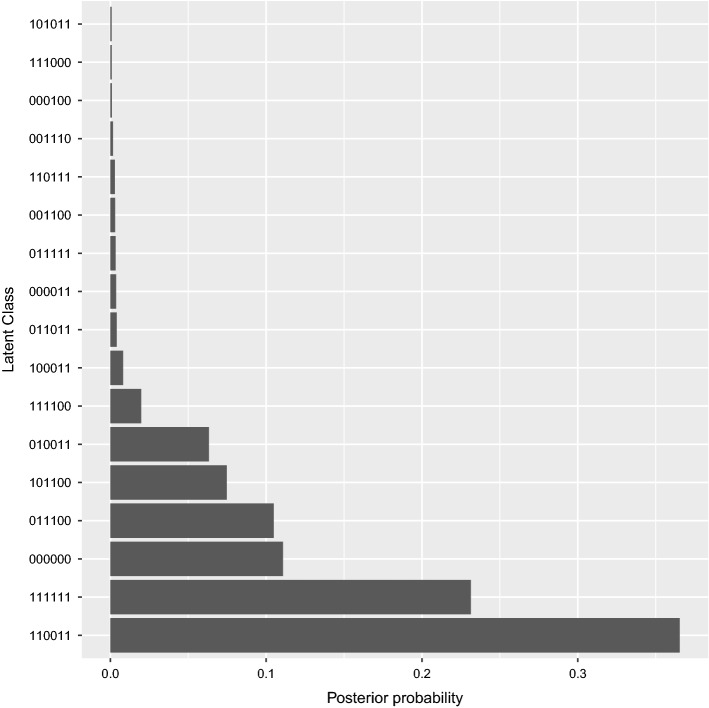
Plot of proportions of latent classes

The ultimate goal of cognitive diagnostic modelling is to classify test takers according to the presence or absence of attributes in a dichotomous model or the degree of presence of attributes in a polytomous model. Figure 3 demonstrates the proportions of the different latent classes based on this hypothetical example. The most common latent class, i.e., the most common group of examinees, was 110011, the one in which the attributes "Clinical diagnosis" and "Treatment” were absent, with more than one-third of the participants. The second most common latent class was when all attributes were present (111111), accounting for slightly more than 20% of the test participants.

**Fig. 4:**
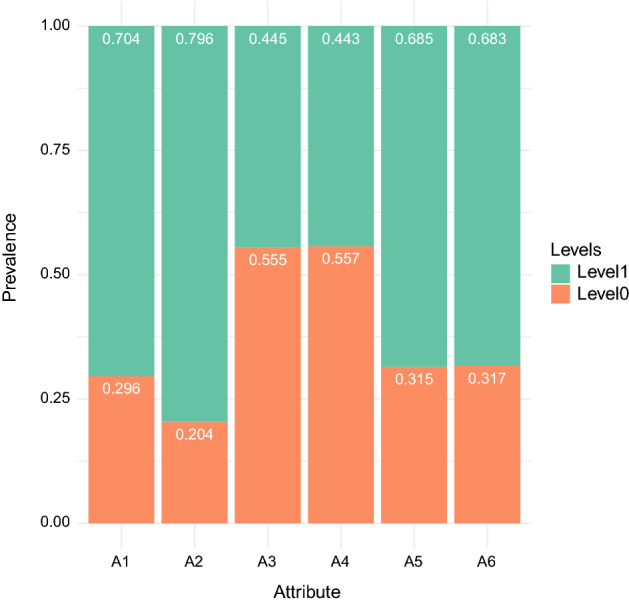
Plot of attribute prevalence

**Fig. 5:**
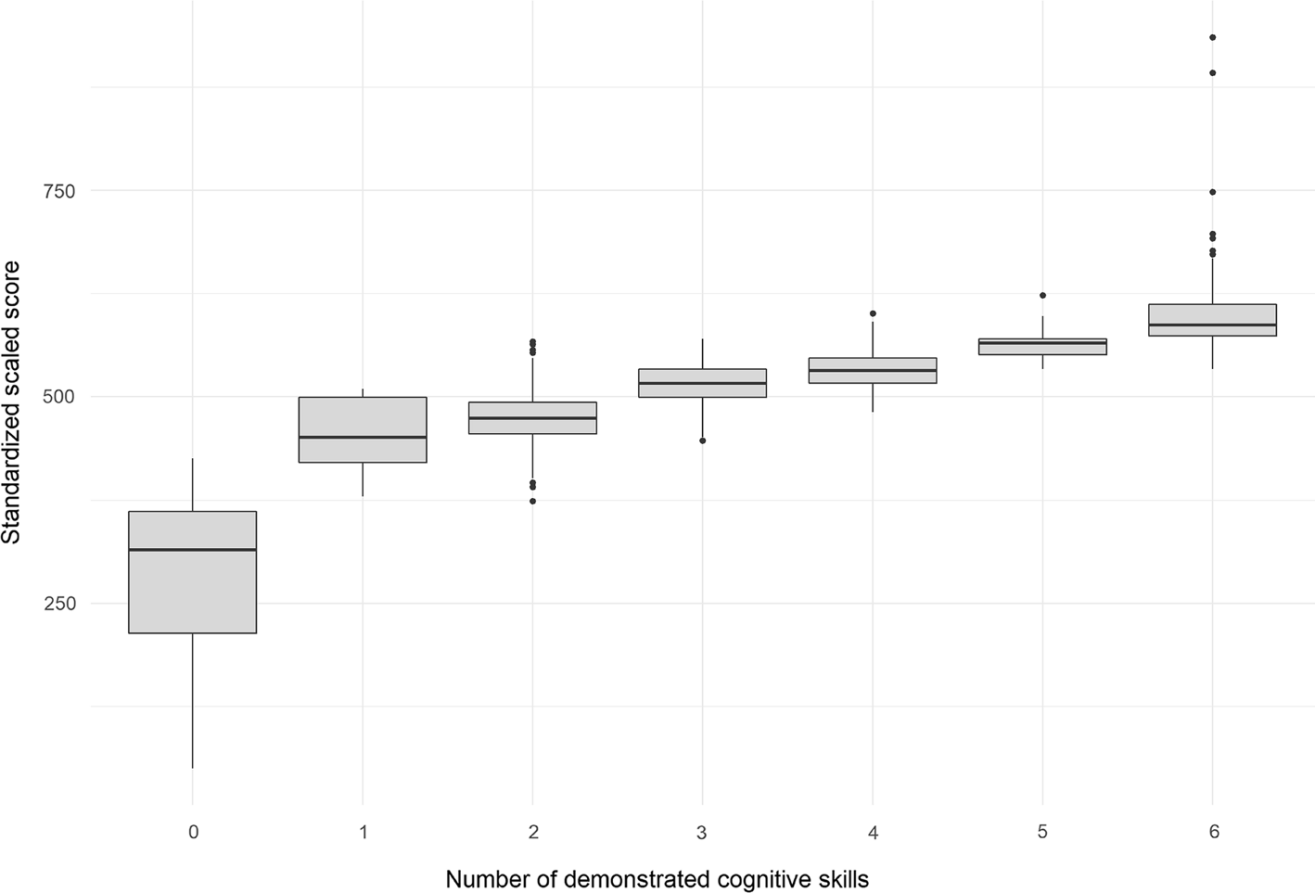
Boxplots of the distributions of proportions of correct responses (standardized to mean = 500 and standard deviation = 100), according to the number of demonstrated cognitive attributes as estimated in the cognitive diagnostic modelling analysis

A closer look at Fig. 4, which shows the prevalence of the attributes separately, reveals that the majority of these hypothetical examinees had mastered all attributes except for the ones that are perhaps the most critical for future professionals, namely, "Clinical diagnosis" (A3) and "Treatment" (A4). Fortunately, this was a hypothetical test with simulated data. However, were this test real, many students in this latent class would have been able to obtain a result higher than the arbitrary cut score of 500, culminating in a "pass" grade even with insufficient mastery of the two most important attributes. This result is observable in the comparison between the number of demonstrated attributes and the standardized scores, obtained as proportions of correct item responses, as shown in Fig. 5.

Tatsuoka believed that understanding knowledge structures and enabling the detailed measurement of complex cognitive attributes should have clear implications and serve as a rational basis for instructional design, formative assessment, and improvement of education in general. In her words, “without this understanding, improvement remains largely a trial-and-error process” (Tatsuoka, 2009). In the next section, I invite the reader to reflect upon the possibilities made available by the addition of cognitive diagnostic modelling to their assessment and instructional design strategies and how it could positively influence remediation strategies and pass/fail decisions. I also ask the reader to consider the appropriateness of current cut scoring methods, which may now appear to be an imperfect measurement of the desired professional standards.

## Exploring the use of cognitive diagnosis in healthcare professions education

Thus far, this paper has highlighted how cognitive diagnostic modelling differs from older psychometric paradigms by classifying test takers according to their mastery or nonmastery of a large number of specified latent categorical attributes. Furthermore, we have explored how cognitive diagnostic assessments represent a feasible possibility of going beyond the mere use of continuous scales of ability levels. I would like to use this section to further illustrate the possibilities and implications of cognitive diagnostic modelling for our field.

One can assume that most multiple-choice test questions currently tend to ask for the correct diagnosis or treatment given a specific clinical scenario. In these cases, one can expect students to perform better on items where the cognitive task involves pattern recognition, which demands deductive reasoning. As an example, I can ask a student to identify which CT finding is most likely to occur in a patient presenting in the emergency room with aphasia and right hemiplegia with sensory loss. In a context where deductive reasoning items are the most prevalent, one can expect that a large proportion of the examinees may have some difficulty performing the cognitive transfer necessary to solve items that demand an inductive reasoning line. Alternatively, this question may be inverted to ask students to state the most likely clinical features a patient may present with if they had a left middle cerebral artery obstruction on a CT scan. Suppose the second item is part of a test where most items cover deductive reasoning. Is the second item worse in quality than the first because of a negative item-total correlation? This is unlikely to be the case, as more balanced assessments with better coverage of authentic professional tasks requiring inductive reasoning are necessary. Inductive reasoning items address a specific cognitive attribute desirable for healthcare professionals: to know what to look for in a clinical examination given a particular finding on imaging or from laboratory results. Should we add cognitive diagnostic modelling to our toolkit to make room for tests covering a broader spectrum of cognitive processes? Most likely, yes.

Despite the limitations of the current hegemonic psychometric approaches, their appropriate use still has considerable positive aspects for all stakeholders involved in educational assessment. This is mainly because numeric scores will likely continue to be the most commonly used method by which stakeholders, including learners, produce performance comparisons. The evaluation of the quality of assessment tools themselves, such as the identification of bias against particular subgroups of students in the form of "measurement noninvariance" or "differential item functioning" (i.e., parameters that are not stable across all subgroups), should in principle require the existence of a continuous latent variable. This task, however, can also be performed using cognitive diagnostic analyses (Liu et al., 2019). The demonstrable absence of such biases against test-taker subgroups serves as evidence of test validity based on internal structure. Nevertheless, as a general rule, I advise institutional stakeholders to never rely solely on psychometric indicators for their operational decisions about the fate of test items. For example, a careful interplay between quantitative and qualitative arguments on collegiate posttest reviews of items that received student complaints tends to deliver the most appropriate decisions.

One might still criticise cognitive diagnostic modelling as a paradigm that is insufficient for use in programmatic assessment frameworks, as it still relies on cross-sectional and nomothetic logic to report students' results instead of being a longitudinal and idiographic approach. More recently, however, longitudinal cognitive diagnosis models based either on latent transition analysis or high-order latent growth models have been developed to support the longitudinal use of cognitive diagnosis in an assessment for learning approaches (Zhan, 2020).

Considering the growing demand for online virtual learning environments as one of the new challenges posed by the COVID-19 pandemic, one could conceive the use of cognitive diagnosis-based activities to inform learning analytics platforms. As a result, online tutoring may become more "intelligent," including the potential use of cognitive diagnostic computerised adaptive testing (Collares & Cecilio-Fernandes, 2019; Wong et al., 2010). In addition to the personalization of item difficulty levels enabled in adaptive tests based on item response theory, cognitive diagnostic adaptive tests could, in principle, also adjust the test blueprint according to the specific needs of each student.

Personally speaking, I believe that the inclusion of feedback based on cognitive diagnosis modelling in test results may provide an essential tool for learners, teachers and other institutional stakeholders, allowing them to address specific gaps in cognitive attributes, going beyond the remediation of content-wise knowledge gaps only. Moreover, another advantage of cognitive diagnostic modelling, regardless of the chosen model, is that by eliminating the need to provide an estimate of performance within a continuum and using only a classification of proficiency with two or a few classes, higher reliability/accuracy estimates are reached with a much lower number of items. In other words, accuracy in cognitive diagnosis tends to be higher than classical reliability estimates such as Cronbach’s alpha or McDonalds’ omega.

How would students pass in a framework of assessment based on cognitive diagnosis? I believe a "pass" qualification would likely be given according to the demonstrated competencies or cognitive attributes and not simply as a function of the obtained score. The number of so-called "false positives" and "false negatives" on an assessment could eventually be better estimated and ultimately lowered. Alternatively, the scores associated with the latent class in which all attributes of interest have been demonstrated could inform a cognitive diagnostic-based cut score. In this regard, I believe that the use of cognitive diagnosis to calculate the cut score of assessment tools may offer a positive contribution to patient safety. In other words, cognitive diagnostic modelling may indeed provide a feasible alternative to the ongoing reality of “*adding apples and oranges*” in summative assessment.

## Limitations, potential pitfalls and directions for future research

It is important to emphasize that cognitive diagnostic modelling is still an emerging psychometric paradigm and that care should be exercised in its implementation. In the first instance, it might be a burdensome and ineffective task to assign cognitive tasks retrospectively, i.e., to create a Q-matrix in the posttest phase, a procedure also known as retrofitting. It is not recommended to pursue retrofitting a Q-matrix on old items, as misspecifications of a retrofitted Q-matrix may generate inaccuracies in the skill mastery profiles. Ideally, the items and the Q-matrix should be created prospectively, in the pretest phase, using expert panels, as this tends to result in fewer suggestions for changes during Q-matrix validation (de la Torre & Minchen, 2014).

Usually, test developers in healthcare professions already describe the purposes of their assessment tools prospectively. However, the description of a model for the latent skills of diagnostic interest and their interactions (i.e., the “rule space”) is usually not part of their endeavours. A recent attempt to better capture the interactions between attributes in cognitive assessment has fortunately been proposed: the attribute hierarchy method. In this method, the Q-matrix is accompanied by additional matrices, such as the adjacency matrix (“A-matrix”) and the reachability matrix (“R-matrix”), which serve to describe hierarchical or sequential interactions between cognitive attributes (Leighton et al., 2004).

As it is a latent variable model, the implicit assumptions of the model still need to be critically analysed, a concern from Schuwirth and van der Vleuten (2006) that cognitive diagnosis intrinsically cannot resolve. Whether the representation of competencies of interest as latent variables is an appropriate or feasible goal is a topic that will likely be explored in decades to come. Among the assumptions that need careful reflection by educators in healthcare are (a) the representation of skills or competencies as latent variables with two or just a few outcomes, (b) that the items are intrinsically multidimensional, (c) that the set of required cognitive skills can be determined with precision and (d) that the rate–response function of how skill attributes add up, interact, or both can be determined (von Davier, 2018).

Another concern is related to ordinal latent variables, which allow student feedback in a global rating scale style. Using a polytomous approach to cognitive diagnosis requires a careful analysis of the domain or construct, allowing different mastery domain levels to follow the complexity of the cognitive tasks at hand (de la Torre & Minchen, 2014). A potential beneficial impact could be a more precise measurement of performance descriptors. Healthcare professions educators would need to be more conscious of exactly what they are looking for in the students they assess and how they intend to do it.

In terms of computational burden, the number of latent classes for dichotomously defined constructs is 2^n^, where n is the number of cognitive attributes. For example, with the specification of ten dichotomous cognitive attributes, 1024 latent classes will be estimated, and computational times remain acceptable. With more than ten cognitive attributes, however, computational effort will increase, and the chances of nonconvergence of the specified model will also increase, making the use of more than ten attributes somewhat unfeasible with currently available hardware.

Given its characteristic of providing skill mastery profiles, cognitive diagnostic modelling appears to be a natural fit for competency-based medical curricula, at least in terms of knowledge-based (sub)competencies in basic and clinical disciplines. Furthermore, the appropriateness of this model for unravelling the underlying cognitive processes that occur during complex clinical reasoning tasks also deserves special attention. Therefore, the development of cognitive diagnostic script concordance tests is another exciting area for future research. Finally, recently developed methods (facets- and hierarchical rater cognitive diagnostic modelling) can incorporate rater effects – a feature that could be explored in objective structured clinical examinations and other practical assessment tools (Li et al., 2020).

## Developing your own cognitive diagnostic models

Despite the legitimate concerns described in the previous section, the possibility of offering a less pixelated, more fine-grained and richer qualitative picture of students’ performance and knowledge gaps still seems worthwhile, at least for formative purposes. Therefore, I strongly recommend the reader try to gain hands-on experience with cognitive diagnostic analysis. All that is required to start is a matrix containing the scored, usually binary responses, of test takers, and a tentative Q-matrix. Two R packages have become popular for cognitive diagnostic analysis: *CDM* (Robitzsch, Kiefer, George, & Ünlü, 2020) and *GDINA* (Ma & de la Torre, 2020). Both packages deliver comparable results with a high level of replicability (Rupp & van Rijn, 2018). The Shiny graphic user interface provided by the GDINA package makes it more suitable for the less experienced reader who might not want to learn how to write scripts in R to run such analyses. I can reassure the readers willing to do their first cognitive diagnostic modelling analysis that they can find instructions on installing and using the GDINA package and its Shiny graphic user interface in a couple of open-access papers (de la Torre & Akbay, 2019; Rupp & van Rijn, 2018). An online interactive course module on cognitive diagnostic modelling is available free of charge for the reader interested in studying this novel psychometric approach in greater depth (Ma & de la Torre, 2019). The package *cdcatR* (Sorrel, Nájera, & Abad, 2021) can be used by those willing to explore the possibilities of cognitive diagnostic adaptive testing.

## Conclusion

Cognitive diagnostic modelling is not perfect, as I am afraid no psychometric approach will ever be. Nevertheless, cognitive-based diagnostic assessments are being continuously developed and seem to be potentially beneficial for the practice of assessment in healthcare professions education. Perhaps it is time for our community to start exploring the possibilities offered by this novel psychometric paradigm within a framework of assessment *of*, *for* and *as* learning in innovative curricula, particularly if aligned with post-positivist and modern constructivist educational paradigms.
